# TRIM9 Determines Sex-Specific β-Amyloid/Cellular Prion Protein/mGluR5 Complex Formation and Pathological Signaling in Alzheimer’s Disease Mice

**DOI:** 10.64898/2026.07.30.741783

**Published:** 2026-08-04

**Authors:** Fatemeh Babaei, Fatemeh A. Panahi, Tash-Lynn L. Colson, Hang Cheng, Mario Tiberi, Rony Chidiac, Stephane Anger, Stephanie L. Gupton, Khaled S. Abd-Elrahman, Stephen S. G. Ferguson

## Abstract

Biological sex is a major determinant of Alzheimer’s disease prevalence, yet the molecular mechanisms underlying sex-specific vulnerability remain largely unknown. Metabotropic glutamate receptor 5 (mGluR5) functions as a co-receptor for β-amyloid (Aβ42) oligomer/cellular prion protein (PrP ^C^ )-mediated pathogenic signaling in males but not females, establishing a sex-dimorphic node in β-amyloid pathophysiology whose regulatory basis is undefined. Using quantitative proteomic analysis, we identify the E3 ubiquitin ligase TRIM9 as a novel mGluR5-interacting protein and a previously unrecognized sex-specific regulator of the Aβ42/PrP ^C^ /mGluR5 complex. TRIM9 selectively associates with mGluR5 in male but not female APP/PS1 mouse brain and is required for mGluR5 to serve as a co-receptor for PrP ^C^ -dependent Aβ42 oligomer binding. Genetic deletion of TRIM9 abolishes Aβ42/PrP ^C^ /mGluR5 complex assembly in male APP/PS1 mice demonstrating that TRIM9 is an essential scaffold for male-specific Aβ42 signal transduction. Loss of TRIM9 in males further reduces β-amyloid pathology by restoring Akt/GSK3β/ZBTB16-dependent autophagic flux, linking disruption of this complex to a defined downstream proteostatic mechanism. Together, these findings establish TRIM9 as a critical molecular determinant coupling male-specific Aβ42/PrP ^C^ /mGluR5 complex assembly to downstream neurodegenerative signaling and β-amyloid pathology. They reveal an unappreciated layer of sex-dependent complexity in mGluR5 pharmacology and identify disruption of the TRIM9/mGluR5 interaction as a potential male-specific therapeutic strategy for Alzheimer’s disease.

**Significance Statement:**

The molecular basis of sex differences in Alzheimer’s disease vulnerability remains unresolved. We show that mGluR5 functions as a male-specific co-receptor for pathogenic Aβ42/PrP ^C^ signaling and identify the E3 ubiquitin ligase TRIM9 as the factor governing this dimorphism. TRIM9 selectively assembles the Aβ42/PrP ^C^ /mGluR5 complex in male brain, and its genetic deletion disrupts complex formation while restoring Akt/GSK3β-dependent autophagic clearance of amyloid. These findings define a sex-specific signaling axis underlying β-amyloid pathogenesis and establish TRIM9 as a candidate target for sex-informed Alzheimer’s therapeutics.

## Full Text Availability

The license terms selected by the author(s) for this preprint version do not permit archiving in PMC. The full text is available from the preprint server.

